# A Tale of Two Biofilms

**DOI:** 10.1371/journal.pbio.1001119

**Published:** 2011-08-02

**Authors:** Kira Heller

**Affiliations:** Freelance Science Writer, Oakland, California, United States of America

**Figure:**
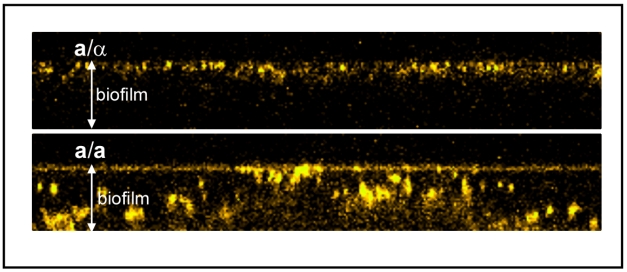
*Candida albicans* produces an asexual (a/α) and sexual (a/a or α/α) biofilm. The former is resistant to penetration by human white blood cells (yellow), whereas the latter is highly penetrable.

One of the great advances in medical technology has unwittingly spawned a serious threat to public health. Implanted medical devices, from cardiac stents to artificial hip joints, are commonly infected with biofilms, complex microbial communities that can prove remarkably resistant to host defenses and treatment. It appears, however, that biofilms, even those arising from the same microbe species, may harbor innate differences in their response to treatments like antifungal agents. Understanding how these microbe colonies might produce structures that appear similar but have very different physical properties and functions is a critical step in figuring out how to overcome antimicrobial resistance. To explore this process, Song Yi, Nidhi Sahni, Karla Daniels, David Soll, and their colleagues turned to the fungal pathogen *Candida albicans*, a major source of hospital-acquired biofilm infections.

In humans, *C. albicans* can cause problems like oral thrush and yeast infections. Far more serious is its increasing tendency to colonize catheters, heart valves, and other medical devices, where it serves as a seeding source for potentially deadly bloodstream infections. *C. albicans* cells have two copies of each chromosome and nearly all strains have different forms of the gene (that is, they are heterozygous) at the genomic region that specifies cell mating-type identity, aptly named the mating-type locus (MTL). These strains are known as **a**/α or MTL-heterozygous. Strains that have the same versions of the gene (either **a**/**a** or α/α) at the mating locus are homozygous. Unlike **a**/**a** and α/α homozygous strains, heterozygous cells can’t make a key transition that’s necessary for mating: they can’t switch from the common white form to the mating-competent opaque form. This is because switching and mating are repressed by a corepressor complex that sits on the MTL of **a**/α cells. Under certain conditions, heterozygous *C. albicans* can become homozygotic and can then make the white/opaque switch.

Like heterozygous cells, white homozygous cells can form biofilms on various surfaces. Previous studies showed that biofilms made of white homozygous cells actually facilitate mating between embedded opaque cells, suggesting that a function of these biofilms may be to conserve mating pheromone gradients. Biofilm formation by homozygous cells is induced by the release of a pheromone that is the first factor in a signal transduction pathway that terminates in the transcription factor Tec1, which binds to regulatory portions of biofilm-related genes. However, heterozygous cells repress pheromone synthesis, indicating that their biofilm formation must be regulated by a different process. To find out more about how these biofilms might be regulated, the Soll lab asked two questions: do the two types of biofilms play different roles in the life cycle of *C. albicans*, and are their biofilms regulated by different signaling pathways?

To address the first question, the authors allowed heterozygous and homozygous biofilms to grow for 48 hours and then examined them under a microscope. They found that all three types (the heterozygous **a**/α, and the homozygous **a**/**a** and α/α) of *C. albicans* formed dense, contiguous biofilms. However, the heterozygous biofilms were about 28% thicker on average than their homozygous counterparts. They also found that dyes with properties similar to antifungal drugs could only penetrate into the upper 15% of heterozygous films, but could penetrate all the way through homozygous biofilms. Furthermore, compared to heterozygous biofilms, heterozygous biofilms were impermeable to a type of white blood cell (called polymorphonuclear leukocytes, or PMNs) that is an important host defense against *C. albicans* infection. In a final permeability test, the authors treated the biofilms with the antifungal agent fluconazole and determined that the heterozygous films had nine times more dead cells than the homozygous biofilms. These results show that heterozygous biofilms are relatively impermeable, suggesting that they function as a protective structure for the cells embedded within them. In contrast, homozygous biofilms, although macroscopically similar, were far more permeable to the white blood cells and less resistant to fluconazole, indicating that their function is less protective and probably has more to do with facilitating mating between embedded opaque cells of opposite mating type.

The researchers explored the pathways regulating the two biofilm types by using different strains of yeast in which a specific gene had been deleted and then measured various parameters such as biofilm adhesion, mass, and thickness. As suspected, they found that the two biofilms are regulated by distinct pathways. Heterozygous biofilms are regulated by a pathway, Ras1/cAMP, known to mediate cell responses to various environmental cues. They also showed that deletion of key components of the Ras1 pathway in homozygous cells did not have an effect on biofilm formation, indicating that homozygous biofilm formation is not regulated by the Ras1/cAMP pathway.

According to Yi and colleagues, regulation of biofilm formation by the pheromone response pathway in homozygous cells evolved relatively recently. Part of this pathway appears to have been co-opted from the pheromone response pathway for mating between opaque cells. However, another part, which comprises Tec1 and its target genes, may well have been modified from the heterozygous biofilm regulation pathway. Unlike the MAP kinase signaling pathway used to regulate biofilm formation in homozygous cells, the Ras1/cAMP pathway does not terminate in Tec1; instead, Tec1 targets another protein called Bcr1. Selective expression of the Bcr1 gene could account for the different properties of the two types of biofilms.

The finding that *C. albicans* can form two different types of biofilm depending on its MTL configuration is interesting not only because it’s an example of how similar structures can have very different functions, but also because it provides information about how signaling pathways may evolve by modifying preexisting signaling modules for entirely new purposes. This phenomenon may be widespread, extending beyond slimy fungal biofilms to a variety of organisms. On a practical note, when researchers are designing new methods to discourage fungal biofilms it will be useful for them to keep in mind that there are two types of biofilms being formed by *C. albicans*.

**Yi S, Sahni N, Daniels KJ, Lu KL, Srikantha T, et al. (2011) Alternative Mating Type Configurations (**a**/α versus** a**/**a **or α/α) of** ***Candida albicans*** **Result in Alternative Biofilms. Regulated by Different Pathways doi:10.1371/journal.pbio.1001117**

